# Characterization of neuroinflammatory positron emission tomography biomarkers in chronic traumatic encephalopathy

**DOI:** 10.1093/braincomms/fcac019

**Published:** 2022-02-03

**Authors:** Cassis Varlow, Ashley C. Knight, Paul McQuade, Neil Vasdev

**Affiliations:** Azrieli Centre for Neuro-Radiochemistry, Brain Health Imaging Centre, Centre for Addiction and Mental Health, Toronto, ON, Canada M5T 1R8; Institute of Medical Science, University of Toronto, Toronto, ON, Canada M5S 1A8; Azrieli Centre for Neuro-Radiochemistry, Brain Health Imaging Centre, Centre for Addiction and Mental Health, Toronto, ON, Canada M5T 1R8; Institute of Medical Science, University of Toronto, Toronto, ON, Canada M5S 1A8; Takeda Pharmaceutical Company, Cambridge, MA 02139, USA; Azrieli Centre for Neuro-Radiochemistry, Brain Health Imaging Centre, Centre for Addiction and Mental Health, Toronto, ON, Canada M5T 1R8; Institute of Medical Science, University of Toronto, Toronto, ON, Canada M5S 1A8

**Keywords:** chronic traumatic encephalopathy, neuroinflammation, PET imaging, TSPO, MAO-B

## Abstract

Chronic traumatic encephalopathy is a neurological disorder associated with head trauma and is confirmed upon autopsy. PET imaging of chronic traumatic encephalopathy may provide a means to move towards ante-mortem diagnosis and therapeutic intervention following brain injuries. Characterization of the neuroinflammatory PET biomarkers, 18 kDa translocator protein and monoamine oxidase-B was conducted using [^3^H]PBR-28 and [^3^H]L-deprenyl, respectively, in post-mortem chronic traumatic encephalopathy brain tissue. [^3^H]PBR-28 displayed high specific binding in both chronic traumatic encephalopathy (95.40 ± 1.87%; *n* = 11 cases) and healthy controls (89.89 ± 8.52%, *n* = 3 cases). Cell-type expression of the 18 kDa translocator protein was confirmed by immunofluorescence to microglia, astrocyte and macrophage markers. [^3^H]L-deprenyl also displayed high specific binding in chronic traumatic encephalopathy (96.95 ± 1.43%; *n* = 12 cases) and healthy controls (93.24 ± 0.43%; *n* = 2 cases), with the distribution co-localized to astrocytes by immunofluorescence. Saturation analysis was performed to quantify the target density of the 18 kDa translocator protein and monoamine oxidase-B in both chronic traumatic encephalopathy and healthy control tissue. Using [^3^H]PBR-28, the target density of the 18 kDa translocator protein in healthy controls was 177.91 ± 56.96 nM (*n *= 7 cases; mean ± standard deviation); however, a highly variable target density (345.84 ± 372.42 nM; *n* = 11 cases; mean ± standard deviation) was measured in chronic traumatic encephalopathy. [^3^H]L-deprenyl quantified a monoamine oxidase-B target density of 304.23 ± 115.93 nM (*n *= 8 cases; mean ± standard deviation) in healthy control tissue and is similar to the target density in chronic traumatic encephalopathy tissues (365.80 ± 128.55 nM; *n* = 12 cases; mean ± standard deviation). A two-sample *t*-test determined no significant difference in the target density values of the 18 kDa translocator protein and monoamine oxidase-B between healthy controls and chronic traumatic encephalopathy (*P* > 0.05), albeit a trend towards increased expression of both targets was observed in chronic traumatic encephalopathy. To our knowledge, this work represents the first *in vitro* characterization of 18 kDa translocator protein and monoamine oxidase-B in chronic traumatic encephalopathy and reveals the variability in neuroinflammatory pathology following brain injuries. These preliminary findings will be considered when designing PET imaging studies after brain injury and for the ultimate goal of imaging chronic traumatic encephalopathy *in vivo*.

## Teaser

Varlow *et al.* report the first *in vitro* evaluation of PET imaging biomarkers for neuroinflammation in chronic traumatic encephalopathy. High variability in neuroinflammatory pathology target density was found in chronic traumatic encephalopathy compared with healthy controls. *In vivo* imaging of neuroinflammatory biomarkers in brain injuries is warranted.

## Graphical Abstract

**Graphical Abstract:**
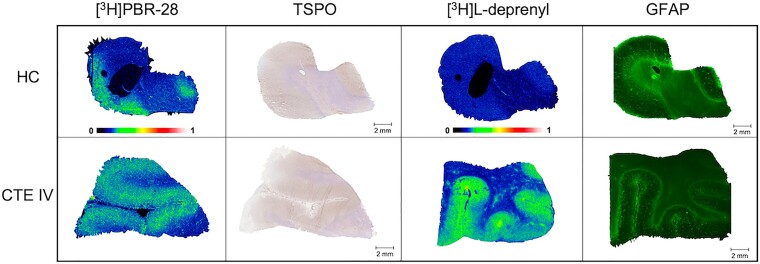

## Introduction

Chronic traumatic encephalopathy (CTE) is a neurodegenerative disease associated with sustained repetitive head injuries. Those at risk for developing CTE include contact sport athletes, military veterans and victims of domestic violence.^1^ Although neurodegeneration can be classified in the clinic by cognitive or behavioural presentations, diagnosis of CTE can only be determined upon post-mortem neuropathological evaluation during autopsy. Diagnosis of CTE is generally based on the perivascular accumulation of hyperphosphorylated tau protein,^2,3^ although the 2014 traumatic encephalopathy syndrome (TES) criteria have been proposed to diagnose CTE *in vivo*.^4^ Revisions of the TES criteria to include cognitive symptoms and application of biomarkers for Alzheimer’s disease have been proposed, as CTE and Alzheimer’s disease share tau aggregation as characteristic pathology.^2,3,5,6^ Without the means to diagnose CTE in life, there are no treatment options for the diverse symptomology and co-morbidities experienced by the impacted individuals.^7–9^ To move towards ante-mortem diagnosis of CTE, PET imaging studies in traumatic brain injury (TBI) populations with tau-targeting radiopharmaceuticals are underway using PET tracers optimized for Alzheimer’s disease-tau including [^18^F]FDDNP, [^18^F]flortaucipir (a.k.a. TAUVID™, [^18^F]AV-1451 or [^18^F]T807) and [^18^F]MK-6240.^7,10–20^ At present, there is no tau-PET radiopharmaceutical optimized for imaging CTE-tau, which can be considered to be mixed 3R/4R tau isoforms.

Neurodegenerative diseases share fundamental pathology including protein aggregation, oxidative stress, apoptosis, autophagal/lysosomal system dysfunction and neuroinflammation. Neuroinflammation is mediated by astrocytes and microglia which become activated/reactive under pathological conditions and response to injury.^21^ It has been hypothesized that microglial activation pathways may provide targets for therapeutic intervention before the clinical effects of recurrent head injury arise.^22^ As such, PET biomarkers have been developed for non-invasive imaging of neuroinflammation. The translocator protein-18 kDa (TSPO) is an outer mitochondria membrane protein and generally considered to be a marker of microglial cells, although non-selective, which is upregulated in response to brain injury and neuroinflammatory disease and is the most studied target for neuroinflammatory processes with PET.^23–25^ [^11^C](*R*)-PK11195 is the first-generation radiopharmaceutical for PET imaging of TSPO; however, its limitations include low signal-to-noise and brain penetration, as well as high blood protein binding.^26,27^ Second- and third-generation TSPO-PET radiopharmaceuticals were subsequently developed, including [^18^F]FEPPA, [^11^C]PBR-28, [^18^F]PBR-06, [^11^C]DPA-713, [^18^F]GE180 and [^11^C]ER176.^25^

PET imaging of TSPO has been conducted in individuals who have sustained TBIs to evaluate short- and long-term neuroinflammation associated with head injuries. In former National Football League (NFL) players, who are considered ‘at-risk’ of developing CTE, a significant increase in total distribution volume (*V*_T_) of [^11^C]DPA-713 to TSPO in 12 brain regions was observed compared with age-matched, healthy controls (HCs).^28^ In a subsequent study using [^11^C]DPA-713, active or former NFL players showed higher *V*_T_ by regional analysis compared with HCs.^22^ However, TSPO expression is not exclusive to reactive microglia but is expressed on reactive astrocytes, endothelial cells and central nervous system infiltrating macrophages and monocytes.^29^ There is also high variability in non-displaceable binding across patient populations that needs to be considered for designing and interpreting TSPO-PET studies.^30^ Another short-coming of TSPO-PET is the sensitivity of second-generation radiotracers to the TSPO single-nucleotide polymorphism (SNP), rs6971, resulting in low-, mixed- and high-affinity binders which requires genetic profiling before imaging with these radiopharmaceuticals.

Alternative biomarkers beyond TSPO are avidly sought to develop PET tracers targeting markers of specific cell types involved in neuroinflammation.^29,31,32^ Monoamine oxidase-B (MAO-B) has been explored as a PET neuroinflammatory biomarker using [^11^C]L-deprenyl-D2,^33^ as this target is primarily expressed on astrocytes as revealed by microautoradiography with [^3^H]L-deprenyl.^34^ Furthermore, in the spinal cord from patients diagnosed with motor neuron disease, MAO-B expression co-localized to reactive astrocytes labelled for glial fibrillary acidic protein (GFAP), a selective astrocyte marker. Increased astrocytic MAO-B activity due to astrocytosis observed in the brains of patients with Alzheimer’s disease may impart oxidative stress through catalytic reactive oxygen species (ROS) generation.^35–37^ Therefore, MAO-B inhibition has been proposed as neuroprotective in diseases, where ROS generation is implicated in neurodegeneration and continues to be investigated.^38^

Our laboratories and others conduct *in vivo* PET imaging studies for pre-clinical and clinical research of TSPO and MAO-B availability to study brain health illnesses including neuroinflammation.^25,39–44^ In the present study, *in vitro* radioligand binding methods were employed to evaluate [^3^H]PBR-28 for TSPO and [^3^H]L-deprenyl for MAO-B to image neuroinflammatory targets in pathologically diagnosed cases of human CTE brains compared with HCs. [^3^H]PBR-28 and [^3^H]L-deprenyl demonstrated optimal specific binding by autoradiography (ARG) and were further characterized for distribution in CTE and validated with immunostaining. Saturation analysis in homogenate tissue was performed to evaluate differences in TSPO and MAO-B target density (*B*_max_) between CTE and HCs. We hypothesize that these experiments will guide *in vivo* PET imaging studies of neuroinflammation in individuals following TBIs.

## Materials and methods

### General

Radiolabelled PBR-28 ([^3^H]PBR-28; 82.0 Ci/mmol (3.03 TBq/mmol), 1.0 mCi/ml (37 MBq/ml)), L-deprenyl ([^3^H]L-deprenyl; 82.0 Ci/mmol, 1.0 mCi/ml), CPPC ([^3^H]CPPC; 79.0 Ci/mmol, 1.0 mCi/ml) and SMW-139 ([^3^H]SMW-139; 82.0 Ci/mmol, 1.0 mCi/ml) were purchased commercially (Novandi Chemistry AB Södertälje, Sweden). Lazabemide hydrochloride (BioTechne, MN, USA) was obtained commercially. All other reagents were purchased from Millipore Sigma unless otherwise stated.

### Human post-mortem brain tissue

Fresh-frozen human CTE and HC brain tissues were obtained from the Boston University-Veteran’s Affairs-Concussion Legacy Foundation Brain Bank, Target ALS Post-mortem Tissue Core and Tissue Solutions in accordance with the guidelines put forth by the Centre for Addiction and Mental Health Research Ethics Board (protocol #036-2019). Tissue demographics are summarized in Table 1.

**Table 1:** Demographic data

Case ID/PIN	Primary pathological diagnosis	Sex	Age
HC(1)	Normal	M	59
HC(2)	Normal	F	63
HC(3)	Normal	M	75
HC(4)	Normal	M	67
HC(5)	Normal	F	57
HC(6)	Normal	M	72
HC(7)	Normal	M	58
HC(8)	Normal	M	48
HC(8)	Normal	F	68
CTE(1)	CTE I	M	30
CTE(2)	CTE I	M	46
CTE(3)	CTE I	M	60
CTE(4)	CTE II	M	46
CTE(5)	CTE II	M	84
CTE(6)	CTE II	M	46
CTE(7)	CTE IV	M	75
CTE(8)	CTE IV	M	74
CTE(9)	CTE IV	M	87
CTE(10)	CTE IV	M	78
CTE(11)	CTE IV	M	71
CTE(12)	CTE IV	M	87

### Genotyping

DNA extraction was performed on all tissue samples (QIAamp DNA Micro Kit, Qiagen) to genotype the rs6971 SNP within the TSPO gene. Genotyping was performed by Agena Mass Array at the Centre for Applied Genomics (The Hospital for Sick Children, Toronto, ON, Canada) to exclude low-affinity binders with Thr/Thr Ala147Thr polymorphism.

### Homogenate preparation

Fresh-frozen human post-mortem CTE (Brodmann area 8/9; superior frontal cortex) and HC (frontal cortex) brain tissues were homogenized to a concentration of 100 mg/ml (wet weight). Tissues were mechanically homogenized on ice in ice-cold 50 mM Tris–HCl buffered saline (TBS) (pH 7.4) using a dounce homogenizer (Fisher Scientific). Homogenates were snap frozen and stored at −80°C.

### Saturation analysis

Saturation analysis of [^3^H]PBR-28 and [^3^H]L-deprenyl in brain homogenates for determination of target affinity (*K*_d_) and *B*_max_ was performed with modifications to previously described protocols.^45,46^ Briefly, frozen aliquots (−80°C) of homogenized (100 mg/ml in Tris-buffered saline, pH 7.4) healthy human brain, or CTE human brain were thawed and diluted 0.25 mg/ml. To define non-specific binding of each [^3^H]PBR-28 and [^3^H]L-deprenyl, unlabelled PK-11195 and lazabemide hydrochloride, respectively, [10 mM in dimethyl sulfoxide (DMSO)], were diluted to 100 µM with assay buffer to yield <1% DMSO/buffer. DMSO concentration was maintained across reactions. Concentrations of [^3^H]PBR-28 and [^3^H]L-deprenyl were increased from ∼0.05 nM to a concentration of at least 10-fold the reported *K*_d_ values.^47,48^ Unlabelled PK-11195 and lazabemide hydrochloride for each [^3^H]PBR-28 and [^3^H]L-deprenyl, respectively, were added to a final concentration of 10 µM within the assay. Reactions were initiated by the addition of brain homogenate to achieve a final concentration of 25 µg tissue/reaction in a final assay volume of 1000 µL. Protein concentration was confirmed by Bradford Assay (Millipore Sigma) and used to normalize saturation analysis. After incubation for 60 min at room temperature, reactions were terminated by filtration over a Whatman GF/B glass fibre filtermat pre-treated with 0.03% polyethylenimine using a Brandel cell harvester (Gaithersburg, MD) and rapidly washed three times with 2 ml ice-cold saline. The filters were soaked in 2 ml scintillation cocktail for 4 h prior to scintillation counting (Tricarb 5110TR scintillation counter; Perkin Elmer). All assays were performed at least in duplicate.

### Autoradiography

Fresh-frozen brains were cryosectioned (Leica CM3050S; 10 µm), thaw-mounted on glass slides (Fisher Superfrost Plus Gold) and stored at −80°C for later use. [^3^H]PBR-28 ARG was carried out to evaluate specific binding, where tissues were incubated with 3 nM [^3^H]PBR-28 with a 10 µM unlabelled PK-11195 or a vehicle in 50 mM Tris/0.9% NaCl pH 7.4 for 1 h at room temperature. [^3^H]L-deprenyl ARG was carried out to evaluate specific binding, where tissues were incubated with 4 nM [^3^H]L-deprenyl with a 10 µM unlabelled lazabemide hydrochloride or a vehicle in PBS + 0.1% BSA for 1 h at room temperature. Tissue sections were subsequently washed with saline (2 × 5 min, 4°C) and deionized water (1 × 10 s, 4°C). Slides were air-dried and exposed to phosphor screens (BAS-IP TR4020; GE Healthcare) for 4 days with a full range tritium standard to permit quantification of radioligand binding (American Radiolabeled Chemicals, Inc.; St Louis, MO). Images were generated by scanning with an Amersham Typhoon phosphorimager (GE Healthcare). Region of interest (ROI) analysis was performed using MCID 7.0 imaging suite (Interfocus Imaging, Cambridge, UK). Raw nanocurie/milligram (nCi/mg), per cent specific binding {% specific binding = [(total signal − non-specific signal)/(total signal)] × 100} is reported.

### Immunohistochemistry

Fresh-frozen human brain tissues were acclimated to room temperature and then exposed to post-fixation by 4% paraformaldehyde. Following a 10 min wash in buffer (TBS containing 0.1% Triton-X), tissues were exposed to protein block (5% goat serum, 0.1% Triton-X 100, 1% BSA; in TBS). Tissues were incubated with rabbit anti-PBR (ab109497; Abcam, Cambridge, MA). After washing, sections were incubated with a rabbit-on-rodent polymer (Vector Laboratories, 30 min) secondary antibody and detected with 3,3′-diaminobenzidine development. Sections were counter-stained with haemotoxylin, dehydrated and coverslipped. Slides were imaged using an Olympus VS200 (Olympus Corporation).

### Immunofluorescence

Fresh-frozen human brain tissues were acclimated to room temperature and then exposed to post-fixation by 4% paraformaldehyde. Following a 10 min wash in buffer (TBS containing 0.1% Triton-X), tissues were exposed to protein block (5% goat serum, 0.1% Triton-X 100, 1% BSA; in TBS). Tissues were incubated with rabbit anti-PBR (ab109497; Abcam, 1:3000), and one of the following: mouse anti-Iba1 (GT10312; Invitrogen, 1:500), mouse anti-CD68 (M0814; Agilent, 1:500), mouse anti-GFAP (G3898; Millipore Sigma, 1:500) or antibody diluent overnight at 4°C and then washed with TBS buffer 3 × 5 min. For detection, sections were incubated with a goat anti-rabbit secondary antibody conjugated to Alexa-Fluor-568 (Invitrogen) and a goat anti-mouse secondary antibody conjugated to Alexa-Fluor-488 (Invitrogen) at room temperature for 60 min. Following washing and 10 min incubation with DAPI, slides were washed with distilled water and coverslipped. Slides were imaged using an Olympus VS200 (Olympus Corporation).

### Statistical analysis

Statistical analyses were performed in GraphPad Prism 8 (San Diego, CA, USA). Statistical significance was evaluated using a two-tailed Student’s *t*-test. No statistical methods were used to predetermine sample size. The sample size was determined by tissue availability.

### Data availability

Data that support the findings of this study are available from the corresponding author upon reasonable request.

## Results

### Specific binding of neuroinflammatory radiotracers in human post-mortem tissue

Initially, we evaluated the tritium-labelled PET tracers [^3^H]PBR-28 and [^3^H]L-deprenyl for TSPO and MAO-B, respectively, and two putative radiotracers/targets for neuroinflammation, namely, [^3^H]CPPC for colony stimulating factor receptor 1 (CSF-1R) and [^3^H]SMW-139 for purinergic receptor 7 receptors. Thin section ARG was performed to evaluate the specific signal of all four radiotracers for their respective neuroinflammatory targets. With previously reported conditions,^49^ [^3^H]SMW-139 at 15 nM displayed negligible signal in fresh-frozen human post-mortem CTE tissues (*n* = 4; data not shown). [^3^H]CPPC was similarly assayed at 10 nM utilizing previous conditions.^50,51^ Although radiotracer binding was observed in post-mortem CTE tissues, it was not specific as defined by homologous blockade (15.34 ± 8.12%; *n* = 4). Because our preliminary ARG studies with [^3^H]SMW-139 and [^3^H]CPPC in CTE tissues showed little-to-no specific binding, these radiotracers were not evaluated further. High specific binding was observed with [^3^H]PBR-28 (3 nM) in both CTE (95.40 ± 1.87%; *n* = 11) and HC (89.89 ± 8.52%, *n* = 3) tissues. High specific binding was also observed with [^3^H]L-deprenyl (4 nM) in both CTE (96.95 ± 1.43%; *n* = 12) and HC (93.24 ± 0.43%; *n* = 2) tissues.

### Distribution of TSPO: [^3^H]PBR-28 ARG and TSPO immunostaining and immunofluorescence

**Figure 1:**
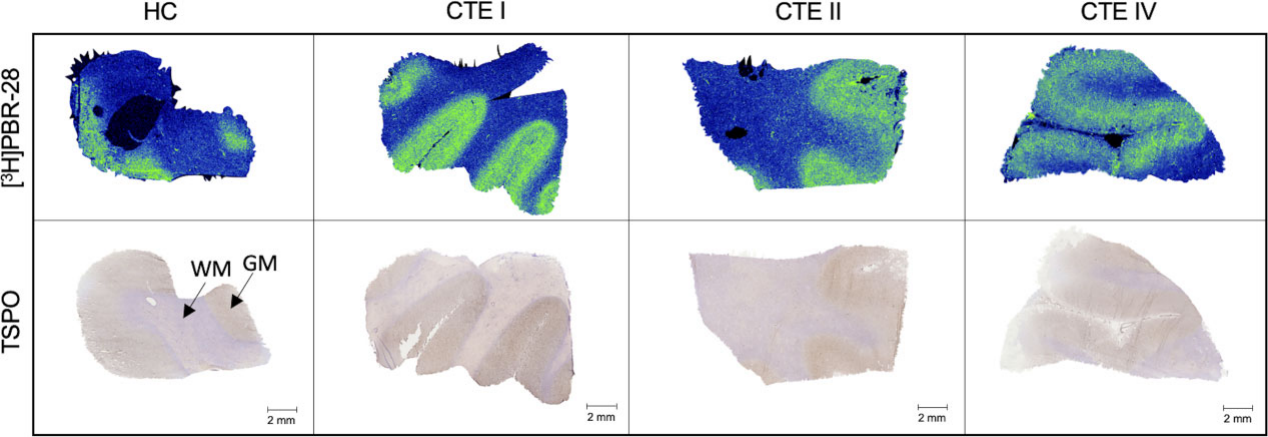
**TSPO distribution in CTE and HC by [^3^H]PBR-28 ARG and TSPO immunohistochemistry.**  *Top row*: Representative images of total [^3^H]PBR-28 (3 nM) signal in HC(5), CTE(2), CTE(5) and CTE(10) post-mortem tissue sections. *Bottom row*: Representative images of TSPO immunohistochemistry on whole tissue sections in HC, CTE I, CTE II and CTE IV at 20×. Scale bar denotes 2 mm.

To characterize [^3^H]PBR-28 distribution in CTE and HC tissues, ARG was performed at a 3 nM radioligand concentration and whole section images were validated by immunohistochemistry with an anti-TSPO antibody (Fig. 1). The ARG signal aligned well with the immunostaining signal, where elevated radiotracer corresponded to areas of more dense antibody staining.

**Figure 2:**
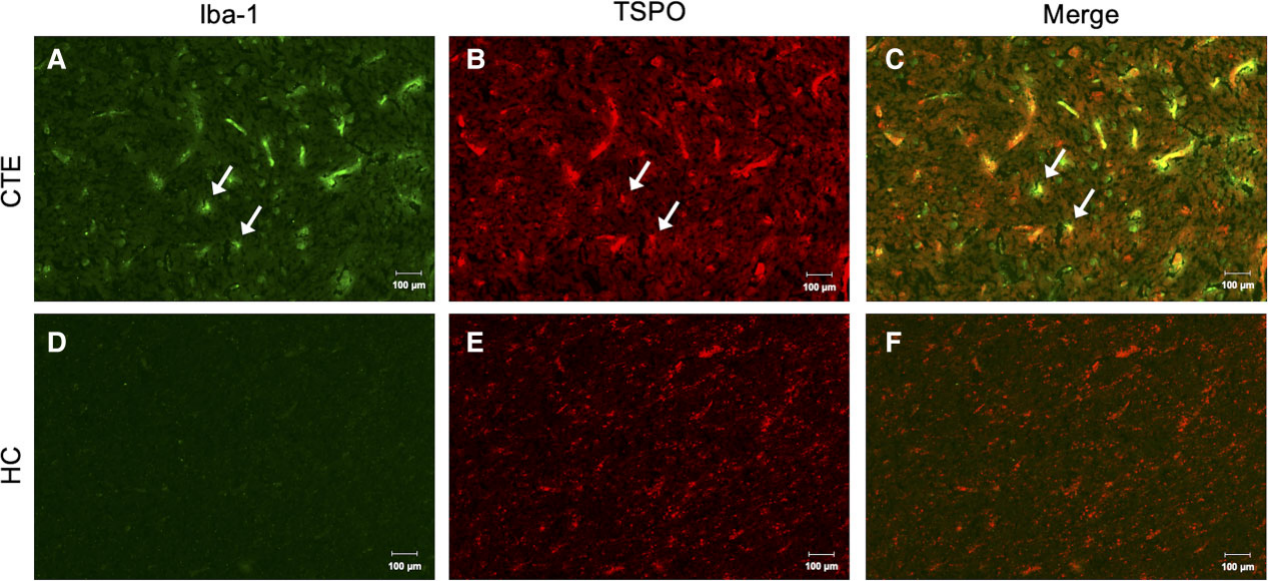
**Immunofluorescent TSPO localization to Iba1 positive microglia.** Representative images of (**A**) immunoreactive Iba1 cells in CTE(2) tissue, (**B**) immunoreactive TSPO cells in CTE(2) tissue, (**C**) merged immunoreactive Iba1 and TSPO cells in CTE(2) tissue, (**D)** the absence of immunoreactive Iba1 cells in HC(5) tissue, (**E**) immunoreactive TSPO cells in HC(5) tissue and (**F**) merged immunoreactive Iba1 and TSPO cells in HC(5) tissue. 20×; scale bars denote 100 µm.

Dual immunofluorescent staining was utilized to show co-localization of TSPO positive cells with cell-type selective antibodies. Iba1 positive staining was observed in CTE tissue (Fig. 2A), along with TSPO positive staining (Fig. 2B). Co-localization of the Iba1 and TSPO signal was also observed, indicating microglial expression of TSPO in CTE (Fig. 2C). In HC tissue, there was little-to-no Iba1 positive signal by immunofluorescence (Fig. 2D); however, there was a positive TSPO signal (Fig. 2E). Co-localization of Iba1 to TSPO is likely attributed to blood-vessel staining, rather than microglial localization of TSPO (Fig. 2F).

**Figure 3:**
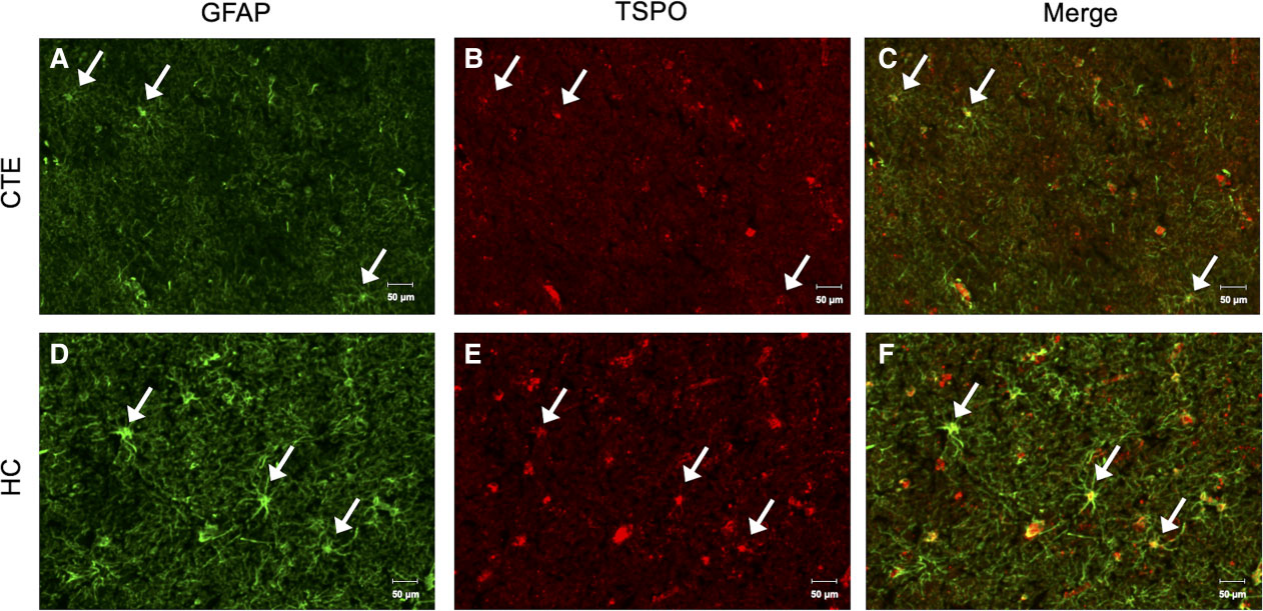
**Immunofluorescent TSPO localization to GFAP positive astrocytes.** Representative images of (**A**) immunoreactive GFAP cells in CTE(4) tissue, (**B**) immunoreactive TSPO cells in CTE(4) tissue, (**C**) merged immunoreactive GFAP and TSPO cells in CTE(4) tissue, (**D**) the absence of immunoreactive GFAP cells in HC(5) tissue, (**E**) immunoreactive TSPO cells in HC(5) tissue and (**F**) merged immunoreactive GFAP and TSPO cells in HC(5) tissue. 20×; scale bars denote 50 µm.

Dual immunofluorescent staining was performed with antibodies to TSPO and the astrocytic marker, GFAP, to investigate TSPO expression on astrocytes. GFAP immunoreactive cells were identified in CTE tissue (Fig. 3A) along with TSPO immunoreactive cells (Fig. 3B) and merging the two stains displayed co-localization of TSPO to astrocytes in CTE (Fig. 3C). In HC tissue, immunoreactive GFAP cells were identified (Fig. 3D) alongside TSPO immunoreactive cells (Fig. 3E) and co-localization of the TSPO signal to GFAP expressing astrocytes was observed upon merging the stains (Fig. 3F).

**Figure 4:**
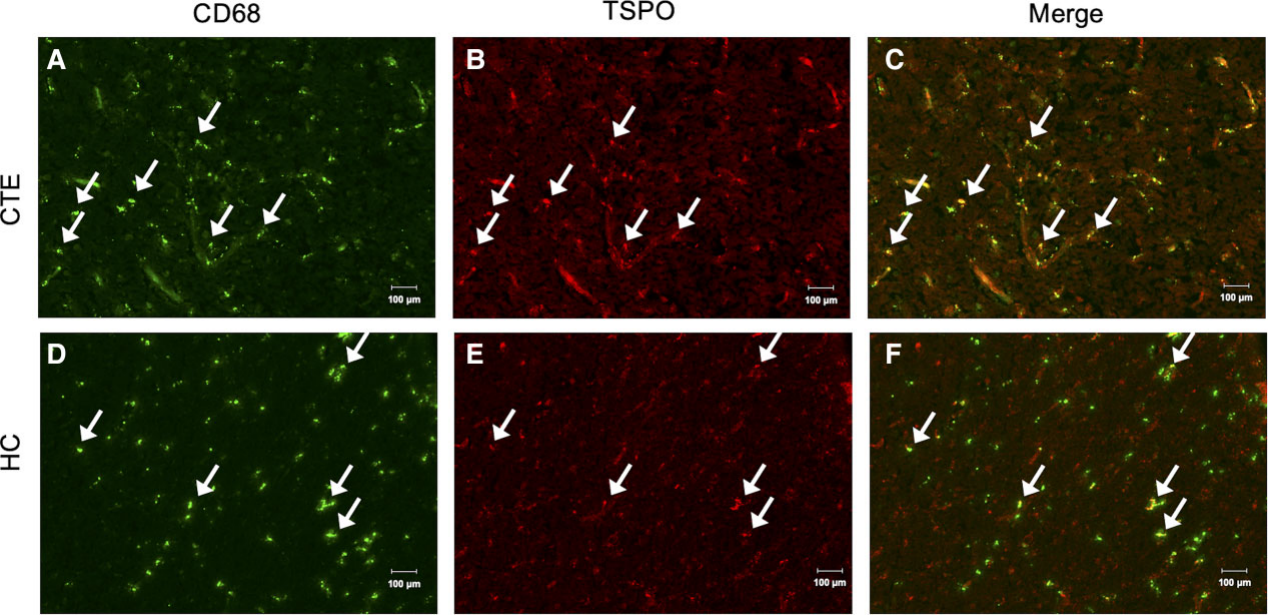
**TSPO immunofluorescent localization to CD68 positive macrophages.** Representative images of (**A**) immunoreactive CD68 cells in CTE(2) tissue, (**B**) immunoreactive TSPO cells in CTE(2) tissue, (**C**) merged immunoreactive CD68 and TSPO cells in CTE(2) tissue, (**D**) the absence of immunoreactive CD68 cells in HC(5) tissue, (**E**) immunoreactive TSPO cells in HC(5) tissue and (**F**) merged immunoreactive CD68 and TSPO cells in HC(5) tissue. 20×; scale bars denote 100 µm.

The last cell-type investigated for TSPO expression was CD68 expressing macrophages and activated microglia. CD68 immunoreactive cells were observed in CTE and HC tissue (Fig. 4A) alongside TSPO immunoreactive cells (Fig. 4B) and co-localization were observed upon merged staining to indicate TSPO expression on CD68 immunoreactive cells. It is of note, however, there is a large cell population of CD68 expressing cells that do not express TSPO.

### Distribution of MAO-B: [^3^H]L-deprenyl ARG and GFAP immunostaining

**Figure 5:**
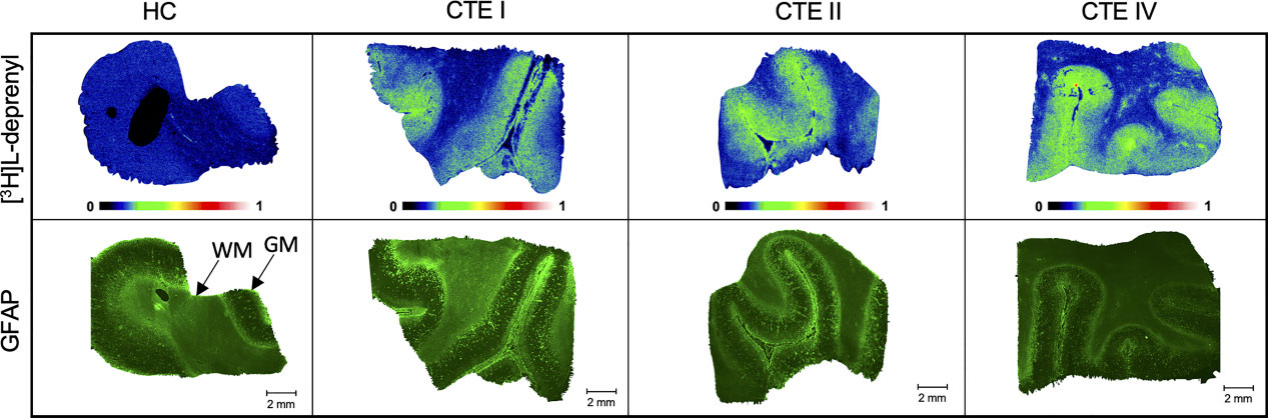
**MAO-B/astrocyte distribution in CTE and HC by [^3^H]L-deprenyl ARG and GFAP immunostaining.**  *Top row*: Representative images of total [^3^H]L-deprenyl (4 nM) signal in HC(5), CTE(1), CTE(6) and CTE(8) post-mortem tissue sections. *Bottom row*: Representative images of GFAP immunofluoresence on whole tissue sections in HC, CTE I, CTE II and CTE IV at 20×. Scale bar denotes 2 mm.

[^3^H]L-deprenyl distribution in CTE and HC tissues was demonstrated by ARG at 4 nM radioligand concentration, and whole section images were evaluated by immunofluorescence with an anti-GFAP antibody to show radiotracer signal corresponding to astrocyte expression (Fig. 5). [^3^H]L-deprenyl ARG signal again aligned well with the immunostaining signal, where elevated radiotracer corresponded to areas of more dense antibody staining.

### TSPO and MAO-B target density in CTE and HC frontal cortex

**Figure 6:**
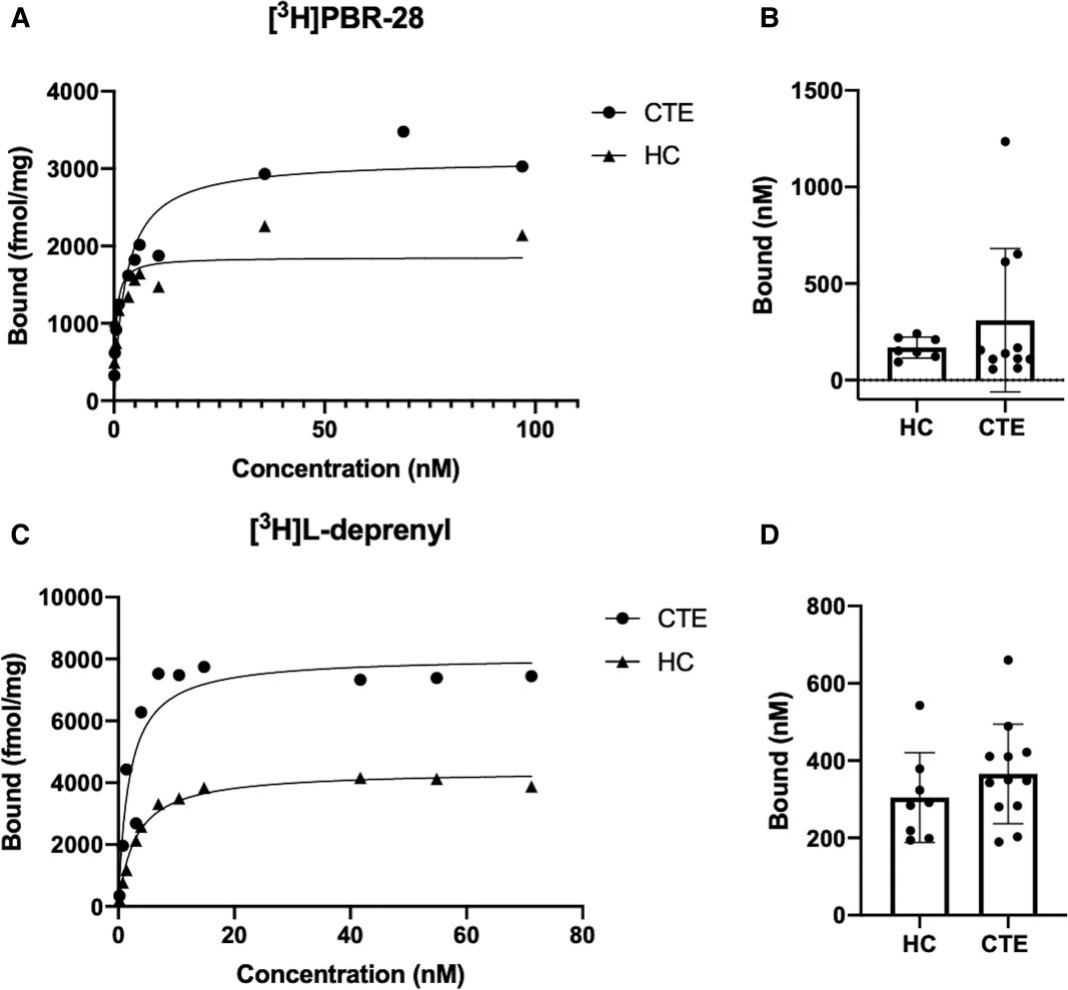
**Target density of TSPO and MAO-B in CTE and HC.** (**A**) Representative saturation curves with increasing [^3^H]PBR-28 concentrations in HC and CTE tissue homogenates. (**B**) Mean TSPO *B*_max_ values in HC tissue (*n* = 7 cases) compared with CTE tissue (*n* = 11) cases. A two-sample *t*-test determined no significance of the differences in TSPO *B*_max_ values between the HC and CTE (*P *= 0.3399, *t* = 0.9838). (**C**) Representative saturation curves with increasing [^3^H]L-deprenyl concentrations in HC and CTE tissue homogenates. (**D**) Mean MAO-B *B*_max_ values in HC tissue (*n* = 8 cases) compared with CTE tissue (*n* = 12 cases). Error bars denote SD. A two-sample *t*-test was performed and no significance was found in MAO-B *B*_max_ values between the HC and CTE (*P *= 0.2903, *t* = 1.090).

Saturation binding assays were conducted to investigate changes in expression of TSPO and MAO-B in CTE compared with HC. Increasing concentrations of [^3^H]PBR-28 (0.1–100 nM) allowed for saturability of the target and a quantification of the *B*_max_. Representative saturation curves are shown for each HC and CTE tissue samples (Fig. 6A). The TSPO *B*_max_ experimentally determined in HC tissue samples was 177.91 ± 56.96 nM [*n* = 7 cases; mean ± standard deviation (SD)] compared with the *B*_max_ in CTE of 345.84 ± 372.42 nM (*n* = 11 cases; mean ± SD; Fig. 6B). A two-sample *t*-test was performed to determine significance of the differences in *B*_max_ between the HC and CTE and no significance was found (*P* = 0.3399, *t* = 0.9838).

To investigate differences in the density of MAO-B in CTE compared with HC, increasing concentrations of [^3^H]L-deprenyl (0.1–75 nM) were incubated with tissue homogenates from HC or CTE samples, and representative saturation curves are shown in Fig. 6C. The MAO-B *B*_max_ was 304.23 ± 115.93 nM (*n* = 8 cases; mean ± SD) in HC tissue compared with 365.80 ± 128.55 nM (*n* = 12 cases; mean ± SD) in CTE tissue (Fig. 6D). To determine if there was a significant difference in *B*_max_ values between HC and CTE, a two-sample *t*-test was performed, and no significance was found (*P *= 0.2903, *t *= 1.090).

## Discussion

### Specific binding of neuroinflammatory targeting radiotracers in human post-mortem tissue

One of the challenges associated with CTE research is the widespread neuropathology across multiple brain regions as the disease progresses.^52^ Tau lesion formation originates in the frontal cortex and increases in size and spreads in later CTE stages and was therefore chosen as the ROI for this study. TSPO expression has been identified on microglia, macrophages, astrocytes, endothelial cells and neurons and is considered to be upregulated under a variety of neuroinflammatory conditions including stimulus and disease.^53,54^ It has been further hypothesized that upregulation of TSPO under inflammatory conditions is attributed to the expression on activated or pro-inflammatory microglial cells, although there is contrary evidence for its downregulation on activated macrophages.^55–57^ MAO-B is primarily expressed on astrocytes in the brain as shown through co-localization to GFAP positive cells by immunostaining.^34^ PET imaging studies have shown increased MAO-B and is attributed to astrocytosis in the brains of Alzheimer’s disease patients and is attributed to astrocytosis,^35–37^ albeit increased MAO-B availability has also been observed in normal ageing.^33^

[^3^H]PBR-28 and [^3^H]L-deprenyl were chosen as tool radioligands for the present work as they have been extensively characterized *in vitro*, for imaging MAO-B and TSPO, respectively.^37,47^ ARG was performed herein to establish *in vitro* signal-to-noise within the HC and CTE tissues for TSPO and MAO-B. Both [^3^H]PBR-28 and [^3^H]L-deprenyl displayed high specific binding in CTE and HC as expected. The high specific binding observed indicated that the present assay conditions were suitable for further target characterization.

### Distribution of TSPO: [^3^H]PBR-28 ARG and TSPO immunostaining and immunofluorescence

[^3^H]PBR-28 was used to establish the localization of inflammatory cells in human post-mortem CTE tissues. TSPO immunohistochemistry was performed to support the distribution of [^3^H]PBR-28 within the tissue sections. ARG studies with [^3^H]PBR-28 showed robust and reproducible specific binding that was preferentially localized to the grey matter, aligning with TSPO immunostaining. The high specific binding demonstrated with [^3^H]PBR-28 allowed for further characterization of TSPO target density in CTE as well as exploring cell-type expression. As TSPO is not localized to a single cell-type, further immunostaining was performed to co-localize TSPO positive cells with cell-type-specific immunohistochemical markers.

Dual immunofluorescent staining was utilized to show TSPO cell-type expression. In CTE tissue, co-localization of Iba1 and TSPO positive signal was observed, indicating microglial expression of TSPO in CTE. However, in HC tissue, there was little-to-no Iba1 positive signal by immunofluorescence in ROIs where there was a positive TSPO signal. Co-localization of Iba1 to TSPO appeared to be attributed to blood-vessel staining, rather than microglial localization of TSPO, and is not unexpected as TSPO is expressed on endothelial cells.^58^ Astrocytic TSPO expression was evaluated by dual TSPO/GFAP staining where co-localized signal was observed in both CTE and HC tissues. Macrophage and activated microglial TSPO expression were evaluated by dual TSPO/CD68 staining with co-localized signal in both CTE and HC tissues.

Although there was TSPO localization to Iba1, GFAP and CD68 positive cells in CTE, there was a prevalent population of TSPO-/Iba1+, TSPO-/GFAP+ and TSPO-/CD68+ cells in each of the samples. This may be attributed to differences in TSPO expression regionally throughout the brain, where the cellular origin of TSPO signal appears to depend on the brain region examined. TSPO signal in the hippocampus reportedly arises from microglia and astrocytes, whereas microglia, macrophages and astrocytes are the main contributors in the substantia nigra.^59^ In this work, evaluation was exclusive to the frontal cortex which may have characteristically unique TSPO cellular origin. These immunostaining studies show that it is imperative to investigate TSPO expression in human tissue acquired from diseased patients compared with controls. Stages of disease progression for CTE represent another variable as neuroinflammatory responses are not uniform, and is consistent with chronic neurodegenerative disease and psychiatric disorders.^60^ The varying cell-type expression of TSPO in CTE demonstrated herein by immunostaining suggests that the contribution of microglia, astrocytes and peripheral infiltrated macrophages all need to be considered when assessing neuroinflammation in CTE with TSPO-PET radiotracers.

### Distribution of MAO-B: [^3^H]L-deprenyl ARG and GFAP immunostaining

Upregulation of MAO-B is a marker of reactive astrocytes and is more cell-type selective than TSPO, suggesting the potential for measuring alteration in MAO-B-PET levels as a marker of astrocytosis in diseases such as Alzheimer’s disease and related dementias.^36^ In CTE, mRNA transcripts associated with neuroinflammation were elevated in CTE astrocyte groups compared with HCs, suggesting that white matter alterations are a critical aspect of CTE neurodegeneration.^60^ Herein, [^3^H]L-deprenyl was used to establish the localization of astrocytic cells in human post-mortem CTE tissues. GFAP immunohistochemistry was performed as MAO-B expression is primarily attributed to astrocytes.^61^ [^3^H]L-deprenyl ARG showed high specific binding that was preferentially localized to the grey matter which aligned with GFAP immunostaining. The high specific binding observed with [^3^H]L-deprenyl allowed for further characterization of MAO-B target density in CTE.

### Quantification of TSPO and MAO-B target density in CTE and HC frontal cortex

Saturation analysis was carried out to quantify the *B*_max_ of TSPO and MAO-B in various stages of CTE frontal cortex compared with HCs. For *B*_max_ quantification, all CTE samples were grouped together to get a single representative value from a larger number of cases rather than several values from smaller subgroups of each CTE stage evaluated. Saturation analysis with [^3^H]PBR-28 quantified a *B*_max_ for TSPO that was elevated in CTE compared with HC. Due to high variability within the CTE samples, the difference in *B*_max_ was not significant. Interpretation of the TSPO *B*_max_ followed clinical practice to include both mixed- and high-affinity binders^62^; however, upon a Grubb’s test to identify outliers within the CTE samples, one mixed-affinity case was identified (*α* = 0.05). This value was not excluded as the high variability in pathology is characteristic of CTE but should be noted upon interpretation of the data. TSPO expression has been reportedly regulated by a variety of factors including immune cell activation state, neuronal activity and ROIs studied.^55,59,63^ The inconsistencies with TSPO expression between neuroinflammatory conditions contribute to the need for additional neuroinflammation imaging targets with higher specificity than TSPO towards CTE.

[^3^H]L-deprenyl quantified the MAO-B *B*_max_ of 304.23 ± 115.93 nM (*n* = 8 cases; mean ± SD) in HC tissue and is similar to the *B*_max_ in CTE tissues (365.80 ± 128.55 nM; *n* = 12 cases; mean ± SD). A two-sample *t*-test determined no significance in *B*_max_ values between HC and CTE for TSPO (*P* = 0.3399, *t* = 0.9838) or MAO-B (*P* = 0.2903, *t* = 1.090). With a recent focus on quantification of astrocytosis in CTE,^60^ we measured differences in expression of MAO-B. Saturation analysis with [^3^H]L-deprenyl quantified the MAO-B *B*_max_ in HC tissue similar to the *B*_max_ in CTE tissues and was not significantly different. There was high variability in MAO-B *B*_max_ within the CTE and HC groups; however, expression of these targets can be impacted by unreported injuries and medications which may contribute to the differences in MAO-B expression observed within the HCs.^64–66^ In addition, no positive correlation between TSPO and MAO-B *B*_max_ values was observed by Pearson correlation (*r* = −0.257, 95% confidence interval from −0.7640 to 0.4428, *R*^2^ = 0.066, *P *= 0.470). Although no significance in *B*_max_ values of TSPO or MAO-B was determined, a trend towards increased TSPO and MAO-B expression was observed in CTE compared with HC.

These findings reveal the variability in pathology of TBIs and challenges associated with the evaluation of CTE and TBI using PET biomarkers for neuroinflammation. Further evaluation of TSPO and MAO-B in CTE is warranted to design PET imaging studies after brain injury and towards the goal of *in vivo* imaging studies of CTE. Further work to explore TSPO and MAO-B expression of CTE in larger sample sizes is needed as post-mortem evaluation of human CTE tissue is required in the absence of representative and reliable animal models. A larger sample size would allow further interpretation of subgroups by genotype and CTE stages. It would also be of value to include additional brain regions for analysis in future studies to explore neuroinflammatory pathology beyond the frontal cortex. While animal models of TBI are used for pre-clinical head injury research, there are caveats with reproducibility and clinical relevance to injuries sustained by humans.^67–70^ Variability in neuroinflammation is expected within CTE as there are many factors to consider in terms of the type of head injuries sustained, the frequency of the injuries, primary anatomical region of impact, duration of sustaining injury, sub-concussive impacts, etc. These factors are being investigated, particularly within current and retired NFL players,^22^ and provide opportunities to understand how head injuries contribute to CTE disease pathology and progression.

## Conclusion

To our knowledge, this work represents the first *in vitro* evaluations of PET biomarkers for neuroinflammation in post-mortem CTE tissue and highlights the variability in neuroinflammatory pathology of brain injuries. PET imaging of patients after TBI in dual tracer paradigms could be used to quantify TSPO and MAO-B and shows promise for achieving the ultimate goal of imaging CTE in the living human brain.

## Abbreviations

CTE =: chronic traumatic encephalopathy
CSF-1R =: colony stimulating factor receptor 1
HC =: healthy control
MAO-B =: monoamine oxidase-B
NFL =: National Football League
ROS =: reactive oxygen species
SNP =: single-nucleotide polymorphism
TSPO =: translocator protein-18 kDa
TES =: traumatic encephalopathy syndrome
TBI =: traumatic brain injury
TBS =: Tris–HCl buffered saline
